# Influence of Impregnation of Recycled Concrete Aggregate on the Selected Properties of Concrete

**DOI:** 10.3390/ma14164611

**Published:** 2021-08-17

**Authors:** Roman Jaskulski, Pavel Reiterman, Wojciech Kubissa, Yaroslav Yakymechko

**Affiliations:** 1 Faculty of Civil Engineering Mechanics and Petrochemistry, Warsaw University of Technology, Łukasiewicza 17 Street, 09-400 Płock, Poland; Wojciech.Kubissa@pw.edu.pl (W.K.); Yaroslav.Yakymechko@pw.edu.pl (Y.Y.); 2 Faculty of Civil Engineering, Czech Technical University in Prague, Thakurova 7, 166 29 Prague, Czech Republic; pavel.reiterman@fsv.cvut.cz

**Keywords:** recycled concrete aggregate, impregnation, concrete durability, compressive strength, freeze-thaw cycles

## Abstract

The paper focuses on investigating the effect of impregnation of recycled concrete aggregate on the mechanical and durability properties of concrete using this aggregate. Cement paste, limewater and diluted water glass were used to impregnate the aggregate. Both a single impregnation and a double impregnation using two different solutions were carried out. A total of four groups of concrete series, with two values of w/c ratio (0.45 and 0.60), were made. Concrete made using the impregnated aggregate was tested and the results were compared with those of concrete made using untreated recycled aggregate of the same kind. The results indicate that impregnation of aggregate improves the mechanical properties of concrete in many cases but reduces its resistance to cyclic freezing and thawing. Furthermore, in the case of impregnation with two solutions, the order in which the impregnants are applied influences the effect obtained. Using the results received, the impregnation methods were ranked in order from best to worst. The best impregnation method proved to be with cement paste, followed by diluted water glass, while the worst results were obtained with limewater, followed by diluted water glass.

## 1. Introduction

The growing environmental awareness of societies is forcing changes in the economy leading to greater use of waste and secondary raw materials. This trend does not bypass the construction industry, including concrete production. Apart from the search for more ecological binders, which does not necessarily mean resigning from the use of Portland cement, aggregates obtained from natural sources are increasingly being replaced. As substitutes, aggregates from waste materials such as metallurgical slags [1,2,3,4], recycled asphalt pavements [5,6], recycled ceramic [7,8], and recycled brick [9,10], as well as aggregates obtained from concrete recycling are being used [11,12].

The latter are probably the most commonly utilized. However, their use is subject to certain restrictions due to less favourable properties of both the aggregate itself and often concrete made with its use [13,14]. In EN 206:2013 [15], the use of recycled concrete aggregate (RCA) in concrete for some exposure classes is excluded completely. The reason for the poorer parameters of recycled aggregate is the high proportion of mortar that is derived from the concrete from which it was obtained. The share of mortar is particularly high in the case of aggregate of fractions below 4 mm, therefore such aggregate is often not used, although attempts are made to apply it after appropriate treatment [16].

The presence of cement mortar in recycled concrete aggregates (RCA) makes the concrete more absorbent, which is associated with increased porosity. Moreover, the transition zone between cement paste and aggregate (ITZ), which already exists in such aggregate, is a weak point, which determines worse results, among others, in the aggregate crushing test. There are three types of ITZs in recycled aggregate concrete: between the aggregate and new cement paste, between the aggregate and old cement paste and between the old and new cement paste. Each of these zones have different properties that further increase the heterogeneity of the material [17,18].

Due to the above-mentioned features of recycled aggregate, its use often involves additional measures to reduce the impact of the RCA’s weaknesses on concrete properties [19]. Three directions of measures can be distinguished here.

The first is the modification of the procedure of concrete manufacturing, or its recipe. These include, among others, preliminary soaking of recycled aggregates, increasing the w/c ratio while maintaining its effective value, or two-stage mixing approach (TSMA) [20] consisting of dosing half the required amount of water to mixed aggregate, adding cement, mixing the components and then adding the rest of the water. A further expansion of TSMA is the triple mixing method (TM) in which, before cement is added, the moistened aggregate is first mixed with pozzolana (fly ash, slag or silica fume) and then cement is added to the mix [21].

The second direction is to clean the aggregate from the adhered mortar. Mechanical cleaning [22,23], chemical cleaning [17,24,25], thermal cleaning [26] and various combinations of these approaches [19,27,28,29] are used.

The third direction is strengthening of adhered mortar [30]. Most often it consists in impregnating RCA with various substances or solutions. The literature contains, among others, examples of application of (poly)vinyl alcohol (PVA) [31], pozzolan slurry [32], emulsion polymer [33], cement slurry [18], silica fume impregnation [34], silicon compounds solutions [35,36] as well as surface improving agents based on mineral oil or silane [37].

An example of a somewhat different approach is introducing CO_2_ into the aggregate [38] or bio-deposition of calcium carbonate [39,40]. This may be a method for strengthening and sealing old, adhered mortar or, on the contrary, for its weakening to facilitate its later removal.

The research presented in this article falls into the area of attempts to strengthen old, adhered mortar in RCA by impregnation. They are a continuation and development of the attempts undertaken earlier by Jaskulski and Mękal [41] which consisted of the impregnation of RCA with water glass. In the following studies, both the set of impregnating materials and the scope of the performed tests were extended. In addition, to analyse the effect of the W/C index on the results, a series of concrete with its two different values were made: 0.45 and 0.6. A novelty is also the execution of a two-stage impregnation using two different impregnants and assessing the influence of the order of their application on the obtained results.

The proposed methods of RCA impregnation can be seen as an extension of the recommendations for the management of construction and demolition waste (CDW) in treatment plants presented in paper [42]. The proposed treatments can be included in the proposed CDW treatment line after stage named “primary treatment” presented in Figure 1 of [42]. The question may arise whether the proposed procedure of two-stage impregnation is feasible under industrial conditions. According to the authors, the answer to such a question is affirmative. However, it is debatable whether to apply a stage of accelerated drying with hot air between the first and the second stage of impregnation in order to maintain the continuity of RCA treatment in the technological process (with simultaneous increase in energy consumption), or to let the aggregate dry naturally, which would entail the necessity of providing some warehouse space for its temporary storage. The latter option would mean stretching the impregnation process over time. It may also require an additional “soft” crushing stage (e.g., using a set of rubberised rollers) before the second impregnation stage. This would be necessary if the aggregate grains were to partially agglomerate during storage. However, it is beyond the scope of this publication to decide which of these solutions would be preferable.

## 2. Materials and Methods

### 2.1. Materials

For the research purposes, 16 batches of concrete were made in two groups of 12 batches and 4 batches, respectively. In all series, quartz sand of 0–2 mm fraction was used as fine aggregate. As coarse aggregate, recycled concrete aggregates were used. They were obtained by crushing concrete rubble from specimens previously made in the laboratory for classes in concrete technology, diploma theses and research work. At the time of crushing, the concrete rubble was at least 6 months old, and the strength of the concrete from which it was made ranged from 30 to 60 MPa. After the crushing, the initially obtained raw aggregate was screened to obtain the target fraction of 4–16 mm. The oversized aggregate was returned to the crusher, and the undersized one was rejected as waste.

For the preparation of all series of concrete, two recipes for each group of series were used. They differed in the value of the w/c ratio, which was 0.45 or 0.60. The same amount of cement, 350 kg/m^3^, was used in all recipes. In the first group of the series, it was CEM I 32.5R, and in the second group, CEM I 42.5N. Both types of cement used were produced by the Ożarów Cement Plant. The recipes used are listed in Table 1.

In addition to the different types of cement, the two groups of the series also differed in the way the mixture was prepared. In the first group, the mixture was made in the traditional way, i.e., first, both aggregate fractions were mixed together, then cement was added, and after mixing the dry components, all the water provided in the recipe was added. In the second group of the series, the mixtures were prepared using a two-stage mixing approach (TSMA). In relation to the original approach described in [19], the proportion of water added in each stage was changed. After mixing, the aggregate was dosed about two-thirds of the required amount of water and the rest (about one-third) after adding cement to the wetted aggregate and mixing them together.

In the case of the 12 series, the recycled aggregate was impregnated by soaking it with a specific solution. Table 2 lists the solutions used for impregnation and the two-letter distinguishing markers of the series impregnated with the given solution. The series marked as GC and GL were soaked first with water glass, and after impregnation with the first solution, were drained and left to dry for about 2 h. The soaking was carried out in a free fall concrete mixer to which air-dry aggregate and impregnating solution were dosed. This process has been divided into three stages. In the stages first and third aggregated was mixed in a mixer for 5 min. In the second stage, the mixer has been stopped for five min. After the impregnation was completed, the aggregate was placed in a thin layer in a laboratory to dry. In order to prevent aggregation of the grains, the dried aggregate was initially raked.

**Figure 1:**
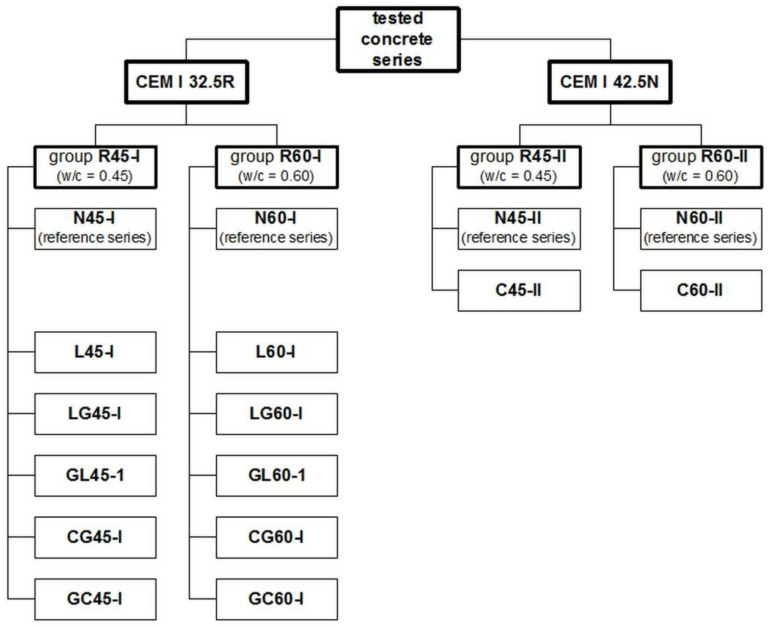
The schematic presentation of the research plan.

The aggregate after the impregnation and drying was stored for two weeks before the concrete was made, except for the C series, where this time was reduced to 1 week. In each group of series listed in Table 1, one reference mixture was made. Aggregate for these mixtures was not impregnated but was rinsed with tap water according to the same procedure as for impregnation. In this way, the possible influence on the results of rinsing the dust fractions from the aggregate during the impregnation process was eliminated. The reference series were marked with N. Because the research plan is quite ramified, so in Figure 1 its scheme is presented.

Each series of concrete consisted of 24 specimens in the form of 100 mm cubes. After preparation of the mixture, its consistency was tested each time using the flow table method as per EN 12350-5:2011 [43]. The results obtained as the consistency class are shown in Table 3.

The concrete mixture was placed in plastic moulds in two layers. Both layers were compacted on a vibrating table. The specimens were de-moulded after two days and then placed in water. The specimens subjected to compression and tensile testing were taken out on the 28th day after the concrete mixture was prepared, and the remaining specimens were still stored in water until they were subjected to the remaining scheduled tests.

### 2.2. Performed Tests

#### 2.2.1. Compressive and Tensile Splitting Strength

Compressive and tensile splitting strength tests were carried out on 28-day specimens in accordance with the provisions of EN 12390-3:2019 [44] (compression) and EN 12390-6:2009 [45] (tension). In both tests, six specimens from each series were used. The tests were carried out using a testing machine with a maximum pressure of 3000 kN at a rate of load growth equal to 0.5 MPa/s for the compressive strength test and 0.05 MPa/s for the tensile splitting strength test. In the second test, spacers made of 10 mm wide hard fibreboard were used.

#### 2.2.2. Free Water Absorption and Sorptivity

Both the free water absorption and later sorptivity tests were carried out on the same halves of 100 mm cubes remaining after the tensile strength test. These specimens were immediately placed in water for three to five days after the strength test. Then they were weighed and placed in a dryer at 110 ± 2 °C for seven days. After that time, the specimens were weighed again and then left to cool down.

The value of absorbability *n* in the percentage of mass was determined using Formula (1),

(1) $$n=\frac{m_{w}-m_{s}}{m_{s}}\cdot 100\%$$

In which *m_w_* is the mass of the saturated surface dried specimen determined immediately after removal from the water, and *m_s_* is the mass of the specimen determined after removal from the oven after seven days of drying at 110 °C.

Sorptivity tests were carried out on cooled halves of cubes using the mass method [46]. For this purpose, the halves were weighed and placed with a flat surface down in a steel tank filled with water in such a quantity that it reached 3–5 mm above the bottom of the specimen. At the established time intervals, the specimens were successively taken out of the water, dried their lower surfaces on a damp towel, and weighed. Immediately after weighing, the specimens were returned to the tank filled with water. Weighing results along with a time marker were recorded by a computer program communicating with the electronic scales. Thanks to this, the exact time of mass measurement was known, which allowed determining the sorptivity value with higher accuracy.

Sorptivity value was determined as a slope of a straight line derived from the mass increments in subsequent measurements in relation to the initial mass, related to the time that passed from the beginning of the test to the time of a given weighing. However, to make this dependence linear, the square root of time was used as an argument for the function to be derived. The linear fitting function is described by Formula (2).

(2) $$\Delta m=S\cdot \sqrt{\Delta t}+b$$

In this formula, Δ*m* is the weight gain of the specimen due to the sorption phenomenon, *S* is the sorptivity value, Δ*t* is the time gain from the start of the test, and *b* is a correction factor that takes into account inter alia disturbances in the capillary suction process. This factor is not interpreted, and its introduction into the equation allows a better fit of the function described by Formula (2). For the same purpose, the procedure for matching function (2) to the results presented in this article does not take into account the starting point where Δ*m* = Δ*t* = 0.

#### 2.2.3. Frost Resistance

The frost resistance test was carried out in accordance with the PN-B-06250:1988 [47] standard. This test determined the drop in the concrete’s compressive strength on specimens subjected to freezing and thawing cycles in relation to the concrete strength tested on specimens of the same age stored in water until the time of testing. For the series of concrete of R45-I and R60-I groups 8 cubes each were used (4 cubes subjected to freezing and thawing cycles and 4 cubes as witnesses). The specimens of these series were between 105 and 120 days old at the time of compressive strength testing. In case of R45-II and R60-II series of concrete, 12 cubes from each series were used (6 cubes frozen and thawed and 6 cubes as witnesses). These specimens were 66 or 70 days old at the time of strength testing. The cubes from all series were subjected to 100 cycles of freezing and thawing.

One freeze-thaw cycle consisted of at least 4 h of keeping the specimens at −18 degrees Celsius (the time was counted automatically from the moment of reaching this temperature in the test chamber) and at least 2 h of thawing the specimens in water at a temperature of about 18 degrees Celsius. Due to the different rate of reaching the set temperature values depending on the amount of inserted material and on the external conditions, the cycle length varied from less than 7 h to almost 8 h. The strength test in the frost resistance test was performed according to the same procedure as described in Section 2.2.1.

The specimens for the frost resistance test were additionally weighed before the very start of the freezing and thawing cycles and before the strength test. The results of these weights were used to determine the correlation between the specimen weight gain and the compressive strength value.

#### 2.2.4. Mercury Intrusive Porosimetry

Pore size distribution was determined by means of mercury intrusion porosimetry (MIP) for series N45-II, N60-II, C45-II I C60-II. The method is based on intrusion of mercury, which is non wetting liquid, to pores of studied sample. The penetrated pore’s diameter is inversely proportional to applied pressure (Washburn equation). The applied pressure is automatically increasing during the experiment and thus mercury penetrates narrower pores. The experiment was carried out by using Pascal 140 and 440 devices (Thermo, Waltham, MA, USA). Pore size distribution has crucial impact on the permeability and thus durability of porous composites [48,49].

Besides pores distribution the permeability of selected mixtures was also computed on the basis of the work of Nokken and Hooton [50], who modified original Katz-Thompson relationship. Derived Formula (3) follows former research dealing with pore characteristics and permeability of cement pastes and concrete [51,52].

(3) $$k=\frac{1}{113}2r_{c}^{2}0.68\eta S\left(0.68r_{c}\right)$$

where *k*—intrinsic permeability (m^2^), *r_c_*—critical pore radius (m), η—total porosity (%), *S*(0.68*r_c_*)—fractional volume of pores larger than 0.68*r_c_*.

## 3. Results and Discussion

### 3.1. Compressive and Tensile Splitting Strength

The results obtained in the strength tests are summarized in Table 4. They are grouped by groups of series. In the first of the three columns with the results, the mean strength calculated from six (or occasionally five) results and the measurement uncertainty are given, based on the number of results and standard deviation using the Student’s t-distribution. The second results column gives the value of the coefficient of variation calculated from the uncertainty of measurement. The last column gives the percentage ratio of the strength value of a given series in relation to the strength value of the reference series.

In order to assess whether the differences between the results obtained for individual series in relation to the reference series are statistically significant, a significance test for mean values was carried out, preceded by the F-test for the equality of variances. In Table 4 the differences of mean values which are not statistically significant are typed in italics.

The first conclusion that emerges during the analysis of the results of the compressive strength test is that the impregnation has had a positive effect primarily on the series of concrete with the higher w/c ratio. In the case of the series of concrete with a w/c = 0.6, the results obtained in five out of six cases of application of the impregnated aggregate proved to be higher compared to the results of the reference series, of which four differences proved to be statistically significant. In the case of concrete series with w/c = 0.45, there were only three such series, and a statistically significant increase of strength occurred only in one series.

Analysing successively the applied impregnation methods, the following relationships can be observed. Limewater proved to be the least effective. Both the L45-I and L60-I series showed reduction of concrete compressive strength by 3.1% and 0.2%, respectively, in relation to reference series. In both cases, however, these reductions are statistically insignificant. The combination of limewater and water glass gave ambiguous results, and the effect depends on the order of application of both impregnating solutions. In the case of LG45-I and LG60-I series, statistically significant differences were obtained, with the former decreasing by 17.1% and the latter increasing by 17.4%. In the case of GL45-I and GL60-I series. On the other hand, an increase in both cases was noted, by 1.9% and 10.5%, respectively. However, only the latter increase was statistically significant. The combination of water glass and cement paste also gave different results and in this case the order of treatment influenced the results. For the GC45-I and GC60-I series, a statistically significant decrease in strength by 6% and a statistically insignificant increase by 2.7%, respectively, were obtained. On the other hand, the CG45-I and CG60-I series showed statistically significant strength increases of 8% and 22%, respectively. A similar pattern of results was obtained for the series with cement paste. Both series C45-II and C60-II demonstrated a statistically significant increase in compressive strength compared to the reference series by 6% and 12.5%, respectively.

The analysis of the results of the tensile strength test indicates very high effectiveness of impregnation treatments. The strength values of series of concrete with impregnated aggregate turned out to be higher in all cases than in the reference series. And only in one case, C60-II series, the difference turned out to be statistically insignificant. The maximum values of strength increase were obtained after impregnating the aggregate first with water glass and then with cement paste, i.e., in case of GC45-I and GC60-I series. The strength increase in both series was 68.1% and 78.2%, respectively. The smallest increase in strength was recorded in the case of impregnation of aggregate with cement paste itself, i.e., C45-II and C60-II series, for which strength increased by 16.1% and 11.2%, respectively. However, if we consider only the results obtained in the R45-I and R60-I group of series, then the lowest increase in strength was recorded in the case of impregnation of aggregate first with water glass and then with limewater. The GL45-I and GL60-I series of concrete made of this aggregate achieved an increase in strength in relation to the reference series by 18.2% and 19.4%, respectively.

### 3.2. Free Water Absorption and Sorptivity

The results of the free water absorption and sorptivity tests are presented in Table 5. Similar to the results of the strength tests discussed earlier, they were also grouped according to the groups of series. The mean values of the measured parameters were calculated on the basis of 10–12 results (outlier results were excluded in some series). Similarly, the measurement error was calculated. As in the case of the strength, the calculated mean values of each series were compared to the mean values calculated for the reference series. The tests determining the statistical significance of the calculated differences were also carried out, marking correspondingly the data in Table 5.

The free water absorption values obtained in the R45-I and R60-I groups of the series indicate moderate, but statistically significant effectiveness of the impregnation procedure in reducing the water absorption of concrete. The decrease of free water absorption value for these series is between 2.7% (GC45-I) and 18.8% (CG45-I). In absolute terms, the drop is between 0.20 (GC45-I) and 1.75 (CG60-I) percentage points. The only series in these two groups with an increase in free water absorption value of 0.3% and 0.02 percentage points was the LG45-I series, but this increase proved to be statistically insignificant. In the case of the series C45-II and C60-II both the decrease in the case of the former and the increase in the case of the latter were small and turned out to be statistically insignificant.

The analysis of the obtained sorptivity results indicates that all the recorded differences are statistically significant. Out of the twelve concrete series in which impregnated aggregates were used, only in three cases an increase in sorptivity, i.e., deterioration of the concrete properties in relation to the reference series, was recorded. All three cases are the series in which the w/c ratio was 0.45 (LG45-I, GC45-I and C45-II). The highest increase in sorptivity, by 32.1%, was observed for LG45-I series. The highest decrease was observed for L45-I series and amounted to 67%. The impregnation with limewater also proved to be very effective in the case of concrete series with w/c = 0.6, i.e., L60-I series, which sorptivity in relation to the reference series decreased by 53.9%. In the remaining cases, the drops in sorptivity values were significantly smaller, not exceeding 20%, with one exception—in the case of CG45-I series the decrease was slightly higher, amounting to 25%.

It is worth noting that in the case of the series with w/c = 0.45, impregnated with two solutions, both in the case of free water absorption and in the case of sorptivity, the influence of the order of application of the impregnating solutions was quite clear. However, in the case of impregnation with limewater and water glass, better results were obtained by using water glass first. On the other hand, in the case of impregnation with water glass and cement paste, better results were obtained when the water glass solution was applied in the second step. It should also be added that out of all series in which two solutions were used for impregnation, the order of their application influenced the results of both free water absorption and sorptivity in a statistically significant way in almost all cases. The only exceptions are the sorptivity values obtained for CG60-I and GC60-I series.

### 3.3. Frost Resistance

**Figure 2:**
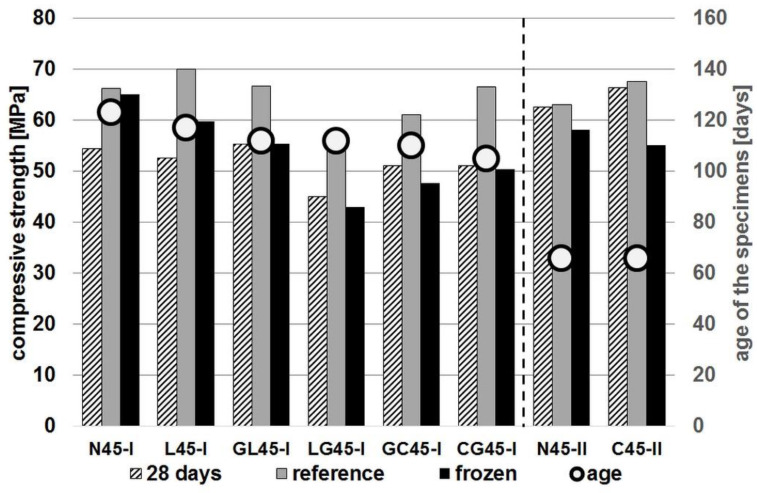
Results of frost resistance test (concrete series with w/c = 0.45).

**Figure 3:**
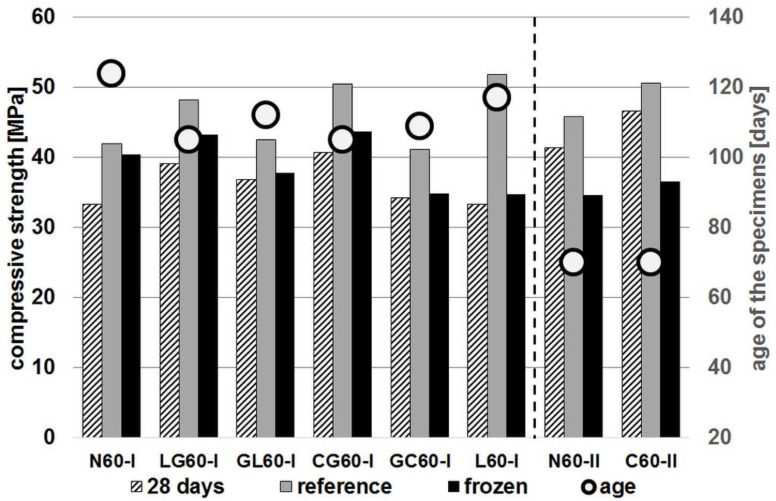
Results of frost resistance test (concrete series with w/c = 0.6).

The results of the frost resistance test are shown in Figure 2 and Figure 3. They compare the compressive strength values obtained after 28 days with the strength values of specimens subjected to freeze/thaw cycles and those not subjected to such cycles serving as a reference. The age of the specimens at the time of the test is also given. In order to facilitate the analysis of the obtained results the concrete series in both drawings are arranged according to the increasing decrease of strength caused by subjecting the material to freeze/thaw cycles. The exception are the concrete series from groups R45-II and R60-II, which are grouped on the right side of each diagram.

The analysis of the results shows that the impregnation of the aggregate has increased the relative strength decreases in all series after the specimens were subjected to freeze/thaw cycles. However, if the criterion of concrete frost resistance according to the PN-B-06250:1988 [47] standard is adopted, i.e., the strength decrease less than 20% in relation to the reference specimens, then it is possible to indicate the series in which this condition has been met. In case of the series of concrete with the index w/c = 0.45, only two series (not counting the reference series N45-I) met this condition. These are the L45-I and GL45-I series, which lost the strength by 14.4% and 17.0% respectively. To this group should be added the C45-II series, for which the strength has decreased by 18.4%.

Among the concrete series with a w/c ratio of 0.6, all series except L60-I, for which the strength has fallen by 33%, met the condition of the strength drop of less than 20%. The N60-II and C60-II series, for which the strength decreased by 24.5% and 27.6%, respectively, also failed to meet this condition. However, it should be remembered that the results of all four series from groups R45-II and R60-II were obtained in tests carried out after about twice as short a time as the series from groups R45-I and R60-I. Therefore, a direct comparison of these groups of series may lead to invalid conclusions.

The general conclusion that can be drawn from the results obtained is that impregnation of aggregate leads to a deterioration of resistance to cyclic freeze-thaw. This effect can be explained by assuming that due to its higher porosity, recycled aggregate provides a kind of reservoir of space for water freezing in concrete. Impregnating the aggregate leads to sealing and reducing access to its pores. This effect is much more pronounced in the case of concrete with a w/c ratio of 0.45. In this case, the impregnation of the aggregate led to a reduction in strength of 81.6%, whereas in the case of untreated aggregate the reduction was only 92.2%. In the case of concrete with w/c = 0.6, the reduction in strength in both cases was comparable: 72.4% and 75.5% for the impregnated and untreated aggregate, respectively. This is probably a result of the recycled aggregate being less saturated with mixing water in concrete with a lower w/c ratio. As a result, more of the pores in this aggregate remained unfilled and could provide additional protection of the concrete structure during freeze/thaw cycles.

Before the start of the concrete frost resistance test and before the subsequent compressive strength test, the weight of the specimens was measured; both those subjected to freeze/thaw cycles as well as the reference specimens. The analysis of the results obtained in this way showed a clear increase in the weight of the specimens subjected to freeze/thaw cycles, as well as an additional clear correlation of this increase with the compressive strength value. In the case of the reference specimens, such a clear increase in mass was not recorded. Also, the change in weight of these specimens did not correlate with the compressive strength value.

**Figure 4:**
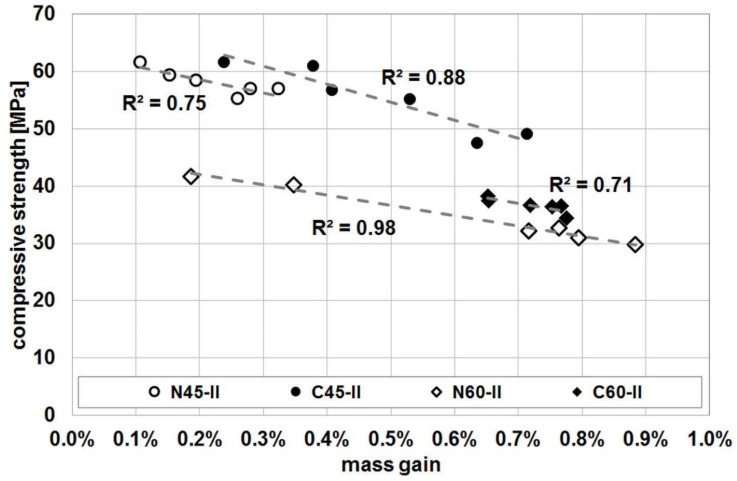
Correlation between specimen weight gain and compressive strength after 100 freeze/thaw cycles.

The obtained relationships between the increase in weight of a single specimen and the obtained value of its strength are shown in Figure 4. They were limited to the results obtained for the N45-II, N60-II, C45-II and C60-II series, because in these cases relationships were based on six results (not four, as in the case of the other series) and thus the obtained R^2^ values are more reliable. It should be noted, however, that the same type of a relationship was recorded for almost all tested series, and the obtained values of R^2^ ranged from 0.79 to 0.98 (significantly higher for a series of concrete with the index w/c = 0.45). Only for series N45-I and N60-I and L60-I such correlation could not be found. In the latter case, the opposite relation was obtained, but it was determined by one result significantly different from the others.

The observed increase in the weight of the specimens can be to a large extent explained by the complete filling of the pores of concrete with water, and even by their “overfilling”, as there was a slight increase in the dimensions of the specimens, and thus their volume. This is the effect of the known phenomenon of sucking water into the pores in the course of successive freeze and thawing cycles. However, the fact that there is such a high correlation between the increase in the weight of a specimen and its compressive strength is intriguing and worth examining more closely.

### 3.4. Porosity

**Figure 5:**
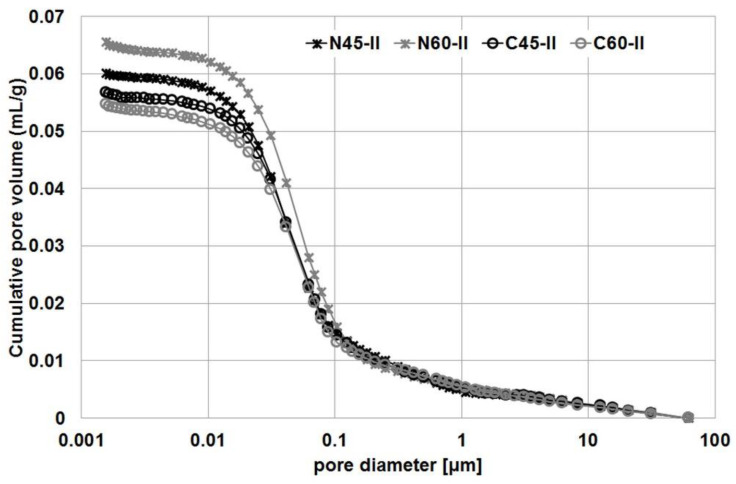
Cumulative pore distribution in R45-II and R60-II series.

**Figure 6:**
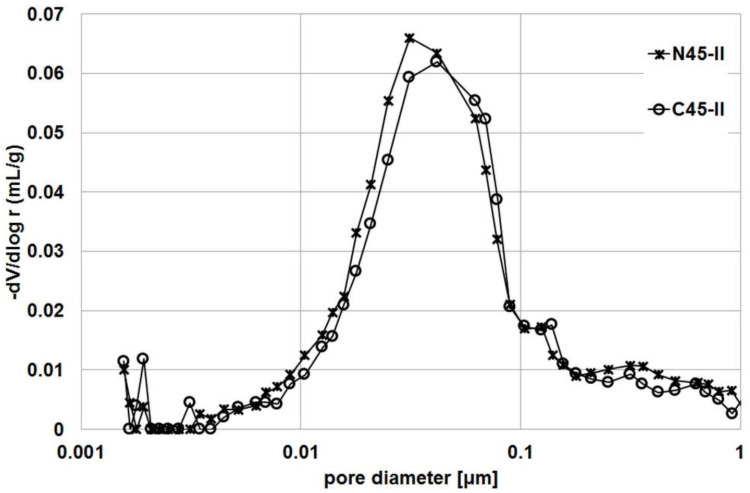
Pore distribution in R45-II series.

**Figure 7:**
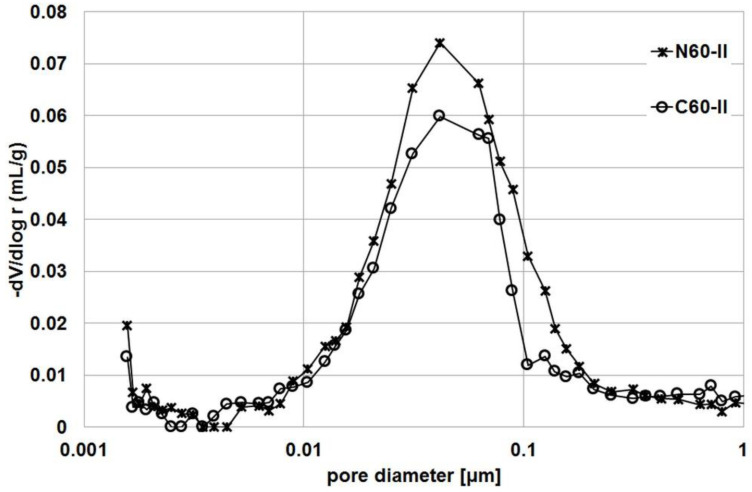
Pore distribution in R60-II series.

The results obtained from the MIP are shown in the Figure 5, Figure 6 and Figure 7. Pore characteristics of selected mixtures were similar to each other, however partial differences were recorded. Mixtures C45-II, C60-II and N60-II exhibited equal values of critical pore radius (*r_c_*), what is well visible in Table 6. However, different values of permeability were derived due to slightly different fractional volumes of used ranges of pore radii. The highest permeability attained mixture N60-II, which exhibited the highest capillary porosity in comparison with other mixtures.

The MIP results clearly indicate that impregnation of aggregate leads to a reduction in the porosity of concrete. A greater reduction in porosity was recorded for the series with w/c = 0.60. The porosity of the series with impregnated aggregate (i.e., C60-II) was 11.64%, while the series of the same composition with untreated aggregate (N60-II) had a porosity of 13.64%. For the series with w/c ratio = 0.45, the porosity values were: 11.90% and 12.61% for the C45-II and N45-II series, respectively.

These results are confirmed by the cumulative pore volume plots in Figure 5, which show clear differences between the series with impregnated and untreated aggregate. A clear decrease in pore volume begins in the 0.1–1 μm diameter range. Figure 6 and Figure 7 compare the pore structure in samples of the R45-II series (Figure 6) and the R60-II series (Figure 6). In order to better illustrate the differences, they were limited to pores with a diameter of less than 1 μm.

As can be seen, the difference in pore volume in both cases covers a similar range of pore diameters. Analysis of the results allows this range to be defined as 0.018–0.078 μm for the series with w/c = 0.45 and 0.021–0.180 μm for the series with w/c = 0.60.

## 4. Summary

In order to comprehensively assess the influence of various impregnation solutions on the change of the properties of the concrete with impregnated recycling aggregate, all the results obtained are summarized in Table 7 in a simplified form showing the qualitative impact of the various forms of the aggregate treatment.

The following markings are used in the table: if the property tested for a given concrete series showed a favourable change in relation to the reference series, then it is assigned the “+” sign, and if the change is unfavourable, then the “-” sign. In addition, the situations in which the change was statistically significant or insignificant were distinguished. In the latter case it was taken in brackets. A slightly different way of assessment was applied in the case of frost resistance. Since the results in all cases were worse than those of the respective reference series, an additional marker was introduced. The sign “o” was used for the series in which the strength drop after 100 freeze/thaw cycles was lower than 20%, which means that the frost resistance condition was met according to the PN-B-06250:1988 [47] standard. The sign “-” meant the strength dropped by more than 20%. No distinction was made for this characteristic between statistically significant and insignificant cases. The symbol used in the case of LG45-I series results from a relatively small exceeding of the frost resistance condition—in the case of this series, the decrease of the strength was 20.4%.

The last two columns in Table 7 contain the total score for each impregnation method calculated assuming that each statistically significant negative or positive effect is assigned the values −1 and +1, respectively. If the effect is statistically insignificant, the values are −0.25 and +0.25, respectively. For the frost resistance, the values 0 and −1 are applied (except for the LG45-I series, where the value −0.25 is given). The values assigned in this way were first summed up, taking into account the first four tested parameters, and the impregnation methods were ranked in the table according to their decreasing efficiency determined in this way. To the results calculated in this way, the values obtained in the frost resistance assessment were added. The supplemented sums are given in brackets. At the bottom of the table, there are also sums of scores obtained separately for concrete series with the w/c = 0.45 and w/c = 0.60.

The analysis of the results obtained, after they were prepared in the manner described above, made it possible to divide the applied impregnation methods into two equally numerous groups. The first one includes methods of high effectiveness. These are impregnations with cement paste and water glass, water glass and limewater (in the given order), and limewater only. The second group includes other cases where the improvement of concrete parameters is not so evident. In their case, the ranking values obtained in Table 6 are also significantly lower and close to each other. A more detailed analysis of these series indicates, however, significant differences in the effectiveness of impregnation treatments depending on the concrete w/c ratio. In two out of three cases (the exception is impregnation with cement paste), much better results were obtained in the case of concrete with w/c = 0.60. The ranking results obtained in these cases are at the level of those calculated for the most effective impregnation methods.

It is worth noting that in all cases the tensile strength of the concrete increased. This parameter can be a good predictor of ITZ quality. An increase in its value means that in concrete with impregnated RCA, the quality of the new ITZ is better than with untreated aggregate. Other concrete parameters examined do not show such a clear improvement, although the free water absorption of concrete with impregnated RCA has decreased in relation to the reference concrete in those cases where the change was statistically significant. Decreased free water absorption indicates a decrease in concrete porosity. This conclusion was partially confirmed by the results of carried out porosity tests.

While water absorption can be used to draw reliable conclusions about the volume of pores accessible to water, sorptivity is derived from their structure. A decrease in the value of this parameter means a deterioration of the conditions of water transport in concrete and, with it, of corrosive factors, i.e., it makes it possible to predict a greater durability of concrete. The results obtained show that sorptivity increased only in the case of three series. And these are only the series with w/c = 0.45. The low value of w/c is in itself a guarantee of the tightening of the concrete structure, so the increase in sorptivity may indicate increased water demand of recycled aggregate. It is worth noting that the increase in sorptivity in relation to the reference series is accompanied by a decrease in compressive strength. This effect is not seen in the case of impregnation with cement paste. However, in this case, the impregnating solution, as a result of the setting process, caused the formation of a durable layer on the surface of the impregnated aggregate. This layer on the one hand sealed the adhered mortar in the RCA and on the other hand probably increased the specific surface area. The higher amount of water absorbed on the surface of the aggregate may have deteriorated the structure of the concrete matrix due to poorer workability, but in this case, there was no decrease in compressive strength, as this adverse effect was compensated by the increase in strength of the aggregate itself.

Speaking of compressive strength, in those cases where its changes were found to be statistically significant, only in the two cases mentioned above was there a decrease. In one case, the aggregate was impregnated with water glass and then with cement paste. Despite an interval of 2 h between the impregnation of both solutions, this order of application of the impregnating solutions did not lead to an increase in the strength of the concrete. The order is crucial here, because the same set of impregnants applied in the reverse order resulted in an improvement of all tested parameters.

An explanation for this effect could be the different characteristics of the two impregnating solutions, which only when applied in the right order produce a synergistic effect. Water glass, although diluted with water, is still a liquid with a much higher viscosity than the cement slurry and was therefore less able to penetrate the mortar adhered to the recycled aggregate. Besides, it does not have binding properties by itself, so it could not form a durable and tight layer on the RCA surface. Its beneficial effect could only come from penetrating and “plugging” part of the bigger pores. Water glass was probably washed out from some of them during the second stage of impregnation. At this stage, the cements did not have the opportunity to penetrate the pores of the aggregate, which had already been filled with water glass. In the case of limewater and water glass impregnation, the mechanism was probably different. The calcium hydroxide, contained in the limewater in much higher concentrations than in the cement paste, reacts with the water glass. The products of this reaction can effectively seal the RCA provided that it is first saturated with the more viscous water glass, which reacts within the pores of the mortar. If the pores are saturated with limewater first, the penetration of the more viscous water glass becomes more difficult, and the reaction mainly takes place on the surface of the mortar.

Analysis of the results of the frost resistance test leads to the conclusion that each of the impregnate combinations used leads to a significant deterioration of the resistance of the concrete to cyclic freezing/thawing. The decreases in the compressive strength of concrete when impregnated RCA is used are significantly greater than for untreated one. This result can be explained by referring to the role played by the pores in concrete in the formation of frost resistance. It is well known that adequate aeration of the concrete increases it. This leads to the conclusion that by impregnating the RCA, and thereby blocking its pores, the concrete is deprived of a certain reserve of free space which can be occupied by freezing water. This leads to the hypothesis that perhaps replacing some of the natural aggregate in the concrete with untreated RCA will have the same effect of increasing the frost resistance of the material as the use of air-entraining admixture. Furthermore, perhaps it will simultaneously reduce the strength loss usually observed for aerated concrete. This hypothesis is already being investigated by the authors and the results of its verification will be presented in one of the planned publications.

It is good scientific practice to compare the results obtained with those reported by other researchers working on similar topics. In the case of the presented study, however, this practice encounters some difficulties. While the literature on various forms of treatment of RCA is very rich, it is difficult to find examples of impregnation carried out in a similar manner as described above. Above all, it was difficult to find examples of two-stage impregnation and with the use of impregnating solutions given above. Some similarities can be found in the method used in the study by Bui et al. [35], where RCA was first soaked in a solution of sodium silicate, dried, and then coated with silica fume. This way of treatment allowed, among others, to obtain an increase in tensile splitting strength by 33–41% and compressive strength by 33–50%. Such an increase is much greater than that obtained in the studies described in this paper, but silica fume is a material with known pozzolanic properties that improve the strength characteristics of concrete.

In the review article by Mistri et al. [30] there is a description and discussion of various methods of RCA impregnation, including some of those described above, but no results of concrete parameter testing. And it is some generally noticeable trend that RCA improvement studies are usually carried out on the aggregates themselves, whose parameter changes are then analysed. In the vast majority of cases, studies of concrete containing such improved aggregate are not carried out. However, if such tests are carried out, they are limited to evaluations of the compressive strength of the material and other characteristics of the material (e.g., porosity) that were not determined in the tests described here. One publication with such results is the work of Ho et al. [31], where the compressive strength and porosity of RCA treated concrete were investigated. The results presented in this work confirm, among other things, the observed reduced effect of impregnation for concrete with lower w/c. In the case of concrete with w/c = 0.4, the applied RCA impregnation caused in some cases a decrease in strength compared to concrete with untreated aggregate. Meanwhile, Martirena et al. [18] reported, among others, the results of a test of the compressive strength of concrete with untreated RCA and RCA coated with cement slurry. After 28 days, they obtained an increase in strength values of 13% when coated RCA was used compared to untreated RCA. The compressive strength of concrete was also studied and presented in the paper by Ismail and Ramli [36]. They used calcium metasilicate and nano silica for impregnation (but without combining both materials). They also obtained results indicating a beneficial effect of RCA impregnation on the compressive strength of concrete.

The above brief literature review indicates on the one hand that the results obtained partly confirm the previously observed effects of RCA impregnation. Partially, since the comparison is limited to the compressive and tensile strengths of concrete. This therefore clearly indicates that this article fills a certain gap in the knowledge of the two-stage impregnation process carried out using cheap, unsophisticated and readily available means. In addition, it complements the existing knowledge with the results of durability parameter tests.

## 5. Conclusions

On the basis of the results obtained, the following conclusions can finally be drawn:

1. The positive effects of the impregnation of recycled aggregate depend not only on the combination of impregnating solutions used but also on the order of their application.
2. The positive effects of the impregnation are much more evident in the case of the concrete series with a higher w/c ratio.
3. All kinds of the impregnations used have improved the tensile strength of the concrete.
4. The free water absorption of concrete with impregnated recycled aggregate has generally decreased in relation to the reference concrete.
5. Sorptivity of the concrete from the series with w/c = 0.60 decreased. In the case of concrete with w/c = 0.45, a decrease was recorded only for half of the series.
6. Compressive strength in six of the series significantly increased related to the reference series, and in two cases, there was a significant decrease. In the other cases, the differences proved to be statistically insignificant.
7. The impregnation with cement paste first, and then water glass, proved to be the most effective in improving the tested concrete properties except for freeze-thaw resistance by w/c = 0.45.
8. The MIP results show a clear reduction in pore volume in concrete with the aggregate impregnated with cement slurry. Although tests were not carried out for the other series, it can be assumed that impregnation with the other solutions produced a similar effect.

## Figures and Tables

**Table 1:** Recipes of tested groups of concrete mixtures.

Content	R45-I	R60-I	R45-II	R60-II
Cement CEM I 32.5R	350	350	---	---
Cement CEM I 42.5N	---	---	350	350
Sand 0–2 mm	608	562	608	562
Recycled concrete aggregate 4–16 mm	1129	1044	1129	1044
Water	158	210	158	210

**Table 2:** Summary of the concrete series designations depending on the impregnating solutions used.

Series	Impregnating Solution (s)
N	none (reference series)
C	cement paste (w/c = 2)
L	limewater (lime:water—1:2)
CG	cement paste + diluted water glass (water glass:water—1:2)
LG	limewater + diluted water glass
GC	diluted water glass + cement paste
GL	diluted water glass + limewater

**Table 3:** Consistency of concrete mixtures according to EN 12350-5:2011 [43].

Concrete Series	Average Flow [mm]	Class of Consistency
N45-I	355	F2
N45-II	410	F2
N60-I	700	F6
N60-II	530	F4
L45-I	380	F2
L60-I	500	F4
C45-II	430	F3
C60-II	448	F3
LG45-I	300	F1
LG60-I	445	F3
GL45-I	325	F1
GL60-I	700	F6
GC45-I	375	F2
GC60-I	700	F6
CG45-I	295	F1
CG60-I	700	F6

**Table 4:** Results of the strength tests.

Concrete Series	Compressive Strength *f_c_*			Tensile Splitting Strength *f_ct_*		
	Tested [MPa]	Coeff. of var. [%]	Relative to the Reference Series [%]	Tested [MPa]	Coeff. of var. [%]	Relative to the Reference Series [%]
group of series R45-I						
N45-I	54.4 ± 1.5	2.81	---	3.86 ± 0.43	11.1	---
L45-I	52.7 ± 2.6	4.88	*96.9*	6.13 ± 0.74	12.1	158.9
LG45-I	45.1 ± 1.7	3.84	82.9	4.91 ± 0.56	11.4	127.3
GL45-I	55.4 ± 2.2	3.95	*101.9*	4.56 ± 0.59	12.9	118.2
GC45-I	51.1 ± 1.8	3.52	94.0	6.49 ± 0.64	9.9	168.1
CG45-I	58.7 ± 1.7	2.91	108.0	5.33 ± 1.06	19.9	138.1
group of series R60-I						
N60-I	33.4 ± 2.0	6.05	---	3.24 ± 0.19	5.9	---
L60-I	33.3 ± 1.5	4.59	*99.8*	4.67 ± 0.50	10.7	144.4
LG60-I	39.2 ± 1.8	4.49	117.4	3.88 ± 0.62	16.0	119.9
GL60-I	36.9 ± 2.3	6.12	110.5	3.86 ± 0.43	11.1	119.4
GC60-I	34.2 ± 0.8	2.28	*102.7*	5.76 ± 0.49	8.5	178.2
CG60-I	40.7 ± 1.4	3.32	122.0	4.55 ± 0.63	13.8	140.6
group of series R45-II						
N45-II	62.6 ± 2.0	3.26	---	3.54 ± 0.43	12.1	---
C45-II	66.4 ± 1.5	2.23	106.0	4.11 ± 0.46	11.2	116.1
group of series R60-II						
N60-II	41.4 ± 0.9	2.20	---	2.92 ± 0.41	14.0	---
C60-II	46.6 ± 1.4	2.94	112.5	3.24 ± 0.29	9.0	*111.2*

**Table 5:** Results of free water absorption and sorptivity tests.

Concrete Series	Free water absorption *n*			Sorptivity *S*		
	Tested [%]	Coeff. of var. [%]	Relative to the Reference Series [%]	Tested [g/(cm^2^·h^0.5^)]	Coeff. of var. [%]	Relative to the Reference Series [%]
group of series R45-I						
N45-I	7.54 ± 0.14	1.86	---	0.112 ± 0.006	6.01	---
L45-I	6.57 ± 0.19	2.89	87.2	0.037 ± 0.004	11.59	33.0
LG45-I	7.56 ± 0.11	1.46	*100.3*	0.148 ± 0.011	7.93	132.1
GL45-I	6.81 ± 0.08	1.17	90.2	0.106 ± 0.013	13.12	94.6
GC45-I	7.34 ± 0.08	1.09	97.3	0.119 ± 0.011	9.66	106.3
CG45-I	6.13 ± 0.22	3.59	81.2	0.084 ± 0.014	17.54	75.0
group of series R60-I						
N60-I	10.00 ± 0.17	1.70	---	0.219 ± 0.007	3.52	---
L60-I	9.45 ± 0.09	0.95	93.8	0.101 ± 0.017	16.73	46.1
LG60-I	8.65 ± 0.19	2.20	85.8	0.186 ± 0.008	4.32	84.9
GL60-I	8.98 ± 0.13	1.45	89.1	0.179 ± 0.013	7.74	81.7
GC60-I	9.49 ± 0.13	1.37	94.2	0.179 ± 0.011	6.21	81.7
CG60-I	8.25 ± 0.22	2.67	81.8	0.183 ± 0.006	3.69	83.6
group of series R45-II						
N45-II	6.65 ± 0.15	2.26	---	0.106 ± 0.006	6.06	---
C45-II	6.64 ± 0.15	2.26	*99.8*	0.110 ± 0.005	4.87	103.8
group of series R60-II						
N60-II	9.12 ± 0.19	2.08	---	0.203 ± 0.004	2.18	---
C60-II	9.19 ± 0.15	1.63	*100.8*	0.179 ± 0.008	4.58	88.2

**Table 6:** Selected results of MIP test.

Concrete Series	*r_c_* (µm)	Porosity (%)	*k* (m^2^)
N45-II	0.0312	12.606	1.335 × 10^−16^
N60-II	0.0417	13.644	2.525 × 10^−16^
C45-II	0.0417	11.898	2.195 × 10^−16^
C60-II	0.0417	11.636	1.433 × 10^−16^

**Table 7:** Simplified qualitative evaluation of the effectiveness of impregnation methods.

Impregnation Method	Concrete Series Group	*f_c_*	*f_ct_*	*n*	*S*	$\frac{f_{c}^{f-t}}{f_{c}}$	Overall-Group Series	Overall—Method
CG	R45-I	+	+	+	+	−	4.00 (3.00) *	8.00 (7.00) *
	R60-I	+	+	+	+	ο	4.00 (4.00) *	
GL	R45-I	(+)	+	+	+	ο	3.25 (3.25) *	7.25 (7.25) *
	R60-I	+	+	+	+	ο	4.00 (4.00) *	
L	R45-I	(−)	+	+	+	ο	2.75 (2.75) *	5.50 (4.50) *
	R60-I	(−)	+	+	+	−	2.75 (1.75) *	
GC	R45-I	−	+	+	−	−	0.00 (−1.00) *	3.25 (2.25) *
	R60-I	(+)	+	+	+	ο	3.25 (3.25) *	
C	R45-II	+	+	(+)	−	ο	1.25 (1.25) *	3.25 (2.25) *
	R60-II	+	(+)	(−)	+	−	2.00 (1.00) *	
LG	R45-I	−	+	(−)	−	− / ο	−1.25 (−1.50) *	2.75 (2.50) *
	R60-I	+	+	+	+	ο	4.00 (4.00) *	
Sum for series with w/c = 0.45							10.00 (7.75) *	
Sum for series with w/c = 0.60							20.00 (18.0) *	

* values in brackets include the assessment of the influence of the impregnation on the frost resistance.

## Data Availability

Data sharing not applicable.
